# Effects of Reynolds and Womersley Numbers on the Hemodynamics of Intracranial Aneurysms

**DOI:** 10.1155/2016/7412926

**Published:** 2016-10-26

**Authors:** Hafez Asgharzadeh, Iman Borazjani

**Affiliations:** Department of Mechanical and Aerospace Engineering, University at Buffalo, The State University of New York, Buffalo, NY, USA

## Abstract

The effects of Reynolds and Womersley numbers on the hemodynamics of two simplified intracranial aneurysms (IAs), that is, sidewall and bifurcation IAs, and a patient-specific IA are investigated using computational fluid dynamics. For this purpose, we carried out three numerical experiments for each IA with various Reynolds (Re = 145.45 to 378.79) and Womersley (Wo = 7.4 to 9.96) numbers. Although the dominant flow feature, which is the vortex ring formation, is similar for all test cases here, the propagation of the vortex ring is controlled by both Re and Wo in both simplified IAs (bifurcation and sidewall) and the patient-specific IA. The location of the vortex ring in all tested IAs is shown to be proportional to Re/Wo^2^ which is in agreement with empirical formulations for the location of a vortex ring in a tank. In sidewall IAs, the oscillatory shear index is shown to increase with Wo and 1/Re because the vortex reached the distal wall later in the cycle (higher resident time). However, this trend was not observed in the bifurcation IA because the stresses were dominated by particle trapping structures, which were absent at low Re = 151.51 in contrast to higher Re = 378.79.

## 1. Introduction

The rupture of intracranial aneurysms (IAs) is highly associated with mortality and morbidity [1]. Hemodynamics has a significant role in the growth and rupture of IAs [2–4]. Among the hemodynamic factors, vortical structures determine the complexity and stability of the flow pattern in an IA dome, which plays an important role in the rupture of IAs [3, 5–7]. Computational fluid dynamics (CFD) holds an important position in the investigation of hemodynamic factors in aneurysms because of its higher resolution near the walls relative to experimental methods such as laser Doppler velocimetry, particle image velocimetry, and magnetic resonance imaging, which is required to compute hemodynamic factors such as shear stress correctly [8, 9]. Many investigations have been carried out on the hemodynamics of IAs using experimental methods [10–12] and CFD [7, 13, 14].

Reynolds and Womersley numbers (explained in Section 3) are the only two nondimensional parameters required for full dynamic similarity in pulsatile internal flows [15]. Therefore, the investigation of their effects on the hemodynamics of IAs is strongly required. However, contradictory conclusions have been made in the literature on this topic. In fact, Jiang and Strother [16] concluded that increase of Womersley number can significantly increase the complexity of the flow pattern and vortex structures in two patient-specific intracranial aneurysms based on their CFD simulations. In contrast, Le et al. [17] stated that Womersley number does not affect the flow feature and structures based on their CFD simulations of an IA from a rabbit. Furthermore, Gopalakrishnan et al. [18] stated that while Womersley number does not change the vortex mode, but high Womersley number is associated with weak vortex rings in their simulation on abdominal aortic aneurysms. Bouillot et al. [10] concluded that Re has a negligible effect on the flow structures of an idealized sidewall intracranial aneurysm for a steady inflow according to their PIV measurements. A similar conclusion was made by Le et al. [17] and Cebral et al. [19] based on their CFD simulations of cerebral aneurysms in rabbits and humans, respectively. In contrast, Gopalakrishnan et al. [18] stated that, by increasing Re, the strength of the main vortex structure increases.

Previous works [7, 19] have shown the significant effect of aneurysm geometry on the aneurysm hemodynamics, which is not investigated further here. Our aim is to compare the effects of the Reynolds and Womersley numbers on the hemodynamics of both sidewall and bifurcations IAs by keeping other parameters, for example, inlet flow waveform and geometry, constant. Furthermore, their effect on the hemodynamics is formulated using dimensional analysis and compared with the literature.

## 2. Governing Equations and the Numerical Method

In this section, Einstein's tensor notation, where repeated indices imply summation, is used unless otherwise indicated (*k*, *d*, and  *q* = 1,2, 3). The governing equations are the 3*D*, unsteady incompressible Navier-Stokes equations for a Newtonian fluid in curvilinear coordinates as follows [20]:

(1) ∂∂ξqUqJ=0,1J∂∂tUm+ξxqm∂∂ξk1JUkuq+∂∂ξk1JPξxqk−1Re∂2∂ξk∂ξd1Jgkduq=0,

where *U* ^*q*^, *ξ* ^*m*^, and *x*_*q*_ are the contravariant velocity, curvilinear coordinate, and Cartesian coordinates components, respectively. *P* and *t* are the nondimensional pressure and time, respectively. *J* is the Jacobian of the geometric transformation, *J* = ∂(*ξ* ^1^, *ξ* ^2^, *ξ* ^3^)/∂(*x*_1_, *x*_2_, *x*_3_), and *ξ*_*x*_*q*__^*k*^ = ∂*ξ* ^*k*^/∂*x*_*q*_ are the metrics of the transformation. *u*_*q*_ is the nondimensional Cartesian velocity, *g* ^*kd*^ is the contravariant metric tensor, *g* ^*kd*^ = *ξ*_*x*_*q*__^*k*^ *ξ*_*x*_*q*__^*d*^, and Re is the Reynolds number of the flow based on characteristic length and velocity scales.

We use curvilinear/immersed boundary (CURVIB) and overset grid methods, which are extensively described and validated [20]. A sharp-interface immersed boundary method is used to handle the 3*D*, arbitrary complex boundaries (IA geometry in this study) inside the flow domain [21]. The nodes that are outside the flow domain are blanked out and do not affect the solution. These nodes are identified using an efficient ray-tracing algorithm [22]. The boundary conditions are reconstructed on the fluid nodes in the immediate vicinity of the immersed boundary along the normal direction to the boundary [21]. The method has been shown to be second-order accurate for a variety of flows [21, 23]. The overset grid approach is implemented to reduce wasted nodes in a domain, which are blanked out by the immersed boundary method [20]. In this approach, a complex flow domain is divided into several arbitrary subgrids with overlaps. To solve the governing equations at each subgrid, boundary conditions at the interfaces are constructed by interpolation from host subgrid. The details of the overset-CURVIB method can be found in [20]. The method has been validated against experimental and benchmark solutions [20, 24] and has been applied to a variety of problems such as cardiovascular flows [17, 25–28], aquatic swimming [23, 29], and rheology [30]. Furthermore, we have validated our method for flows inside an immersed body by comparing our results with the measurements of the pulsatile flow through a 90° bend in Appendix. As shown in Appendix, the computational results are in excellent agreement with our previous simulations using body-fitted grids [20] and the experimental results [31].

We have assumed rigid walls similar to previous simulations [2, 14, 17, 19] because the displacement of aneurysm's wall is typically small and the flow patterns of small distensible and rigid models in the carotid region are very similar [32]. In addition, we have assumed Newtonian fluid in our simulations because the non-Newtonian effects are negligible in larger (>500 *μ*m) arteries [33] and previous simulations of both Newtonian and non-Newtonian fluids have shown similar flow patterns [19].

## 3. Description of Simulated Test Cases

**Figure 1:**
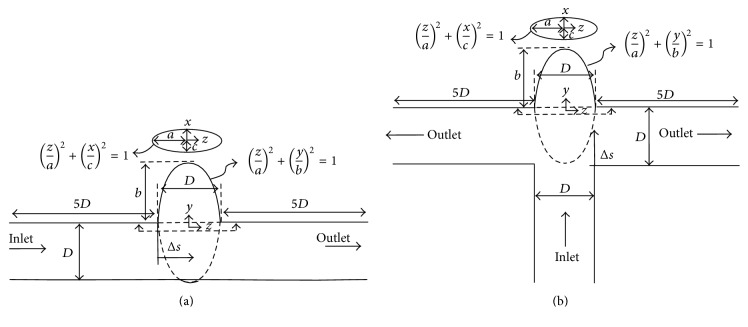
Schematic illustrating of IA simplified models (a) sidewall and (b) bifurcation IA, where ellipse radiuses are *a* = *D*/2, *b* = *D*, and *c* = 0.6*D*). Δ*s* denotes the location of the vortex ring from the proximal wall and inlet/outlet cross section in the sidewall and bifurcation IA, respectively.

Numerical simulations have been carried out on two simplified geometries (sidewall and bifurcation), which can be considered as simplified models of IA, and a patient-specific IA geometry.  Figure 1 shows the configuration of the simplified models for (a) sidewall and (b) bifurcation IAs. The inlet and outlet of the geometries are constructed from a pipe with the diameter *D*. The aneurysm dome is modeled by a cone-like shape with elliptical base and locus, whose radiuses are *a* = *D*/2, *b* = *D*, and *c* = 0.6*D*.

**Figure 2:**
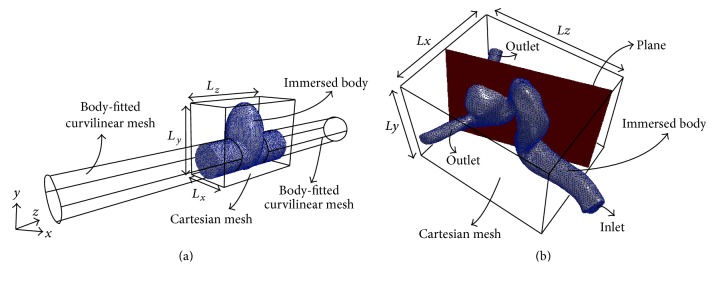
Immersed body and overset grids layout for (a) sidewall IA simplified model and (b) patient-specific IA geometry. The immersed body is meshed with triangular elements.

Figure 2(a) shows the immersed body and overset grids layout for sidewall type IAs and Figure 2(b) shows the immersed body and a Cartesian grid layout for the patient-specific IA geometry. The inlet and outlet(s) in simplified models are meshed by body-fitted curvilinear grids; the circular base is meshed by 41 × 41 grid nodes and the axial (flow) direction is meshed by 81 grid nodes. The geometry of the aneurysm is placed as an immersed boundary onto the uniform grid, and all subdomains (Figure 2(a)) are solved simultaneously [20]. The size of the domain that contains the immersed body is *L*_*x*_ = 1.4*D*, *L*_*y*_ = 2*D*, and *L*_*z*_ = 3*D* and *L*_*x*_ = 1.4*D*, *L*_*y*_ = 3*D*, and *L*_*z*_ = 3*D* for sidewall and bifurcation simplified IAs, respectively. The uniform grid is meshed by 73 × 109 × 153 and 73 × 153 × 153 nodes for sidewall and bifurcation simplified IAs, respectively. Such resolution was found to be enough to obtain grid independent solutions based on our extensive grid refinement studies. Furthermore, the results obtained using a similar resolution for the pulsatile flow through a 90° bend show good agreement with experimental results (Appendix). The size of the domain that contains the immersed body of a patient-specific geometry is *L*_*x*_ = 4.64*D*, *L*_*y*_ = 4.04*D*, and *L*_*z*_ = 5.8*D*, where *D* ( = 0.0048 m) is the hydraulic diameter of the cross section of the inlet vessel and Cartesian grid layout. The uniform grid for the patient-specific geometry is meshed by 233 × 201 × 289 nodes, which has a similar grid resolution with the simplified IAs.

**Figure 3:**
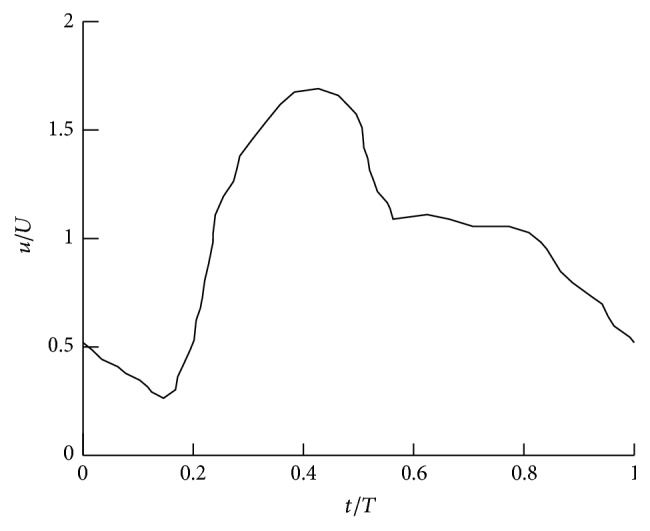
The inlet velocity waveform during one heartbeat. Adapted from [34].

The Neumann boundary condition is applied at the outlet boundaries, and the volumetric flow rate of each outlet is calculated based on the principle of optimal work [36]. The inlet boundary condition is a uniform (plug) flow in space but pulsatile in time with a waveform as shown in Figure 3 with pulsatility index PI = Δ*U*/*U* = 1.43 (where Δ*U* is the difference between the peak systolic and minimum diastolic velocities). The pulsatile velocity waveform is from a patient with a cerebral aneurysm [34]. The numerical simulations were carried out for three cycles to obtain quasi-steady results, which are not affected by the initial condition.

The Reynolds number is defined as Re = *UD*/*ϑ*, where *U* is the average bulk inlet velocity and *D* = 0.005 m and 0.0048 m are the inlet diameter for simplified IAs and the patient-specific IA, respectively, and *ϑ* = 3.3 × 10^−6^ m^2^ s^−1^ is the blood kinematic viscosity (calculated based on *ρ* = 1056 kg m^−3^ and *μ* = 0.0035 kg (ms)^−1^, where *ρ* and *μ* are the blood density and dynamic viscosity, resp. [7]). The Womersley number is defined as $Wo=D\sqrt{2\pi /T\vartheta }$, where *T* is the period of the waveform calculated based on the heart rate. The simulations on the sidewall and bifurcation IAs are carried out with two Womersley and two Reynolds numbers. The Womersley number is modified by changing the heart rate and Reynolds number is modified by altering the inlet bulk velocity in the physiologically relevant range [37]. The nondimensional time-step ($\Delta \bar{t}$) is calculated by dividing the nondimensional *T* into 3200 equal time instants for all simulated cases.  Table 1 shows the simulated cases, their specifications, and denoted names. It should be noted that the calculated Re and Wo in Table 1 are within the physiological range in IAs (173 < Re < 914 and 5 < Wo < 30 [7, 14, 38]).

An (aneurysm number) in Table 1 is the ratio of the transport to vortex formation time scales in IAs [17]. The transport time scale in sidewall IAs is the time it takes for the parent artery flow to transport a fluid particle across the IA neck and the transport time scale in bifurcation IAs is the time it takes for the inlet artery flow to transport a fluid particle to the outlets. An can be defined as follows:

(2) $$\begin{array}{c} An=\alpha \frac{W_{mod}}{D}PI, \end{array}$$

where *W*_mod_ is the aneurysm neck and the outlet diameter in sidewall and bifurcation IAs, respectively, defined based on the transport time scale. *α* = 1 and 2 is determined based on the transport time scale definition for sidewall and bifurcation IAs, respectively. If vortex formation time scale is smaller, that is, An > 1, the vortex is formed before it is advected out of the dome area. In contrast, if An < 1 the flow is dominated by a stationary shear layer from the proximal wall to the distal wall through the IA neck.

## 4. Results and Discussion

In this section, the effects of Reynolds and Womersley numbers on the flow patterns and vortical structures of IAs are investigated. Because of the substantial difference between vortical structures in sidewall and bifurcation IAs, they are investigated in different sections, that is, Sections 4.1 and 4.2 for sidewall and bifurcation IAs, respectively. In Section 4.3, whether observed effects of Reynolds and Womersley numbers (on flow patterns and vortical structures) in simplified IAs can be seen in a patient-specific (complex) IA is explored. In Section 4.4, the location of the vortex ring in simplified IAs and the patient-specific IA is investigated using the nondimensionalization method. Finally, the effect of Re and Wo on the oscillatory shear index and wall shear stress of all test cases is investigated in Section 4.5. The three-dimensional vortical structures are visualized by the isosurfaces of *q*-criteria [35].

### 4.1. Sidewall IA Simplified Models

**Figure 4:**
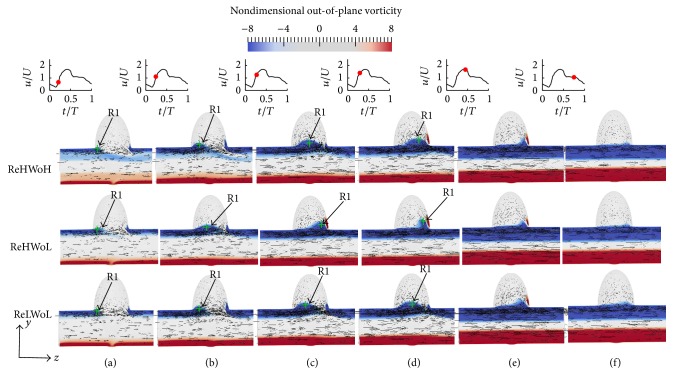
Evolution of the nondimensional out-of-plane vorticity and in-plane velocity vectors for simplified sidewall IAs with different Re and Wo; (upper row) Re = 378.79 and Wo = 9.96; (middle row) Re = 378.79 and Wo = 7.71; and (lower row) Re = 151.51 and Wo = 7.71 at various time instants during the cycle: (a) *t*/*T* = 0.21, (b) *t*/*T* = 0.24, (c) *t*/*T* = 0.27, (d) *t*/*T* = 0.29, (e) *t*/*T* = 0.44, and (f) *t*/*T* = 0.75. R1 depicts the vortex ring in simplified sidewall IA and the green cross shows the location of the maximum *q*-criteria [35] on the midplane of IAs.

**Figure 5:**
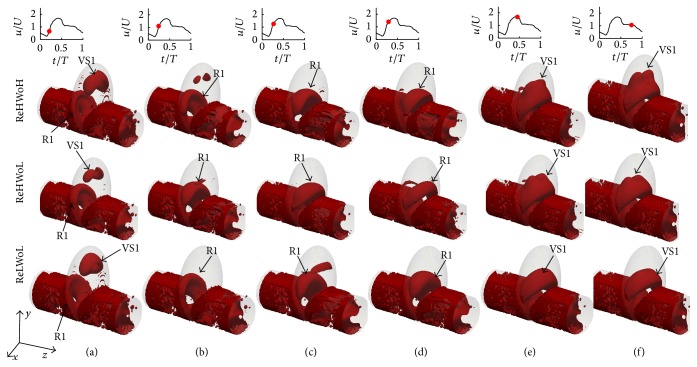
Evolution and topology of the three-dimensional vortical structures for sidewall IAs with different Re and Wo; (upper row) Re = 378.79 and Wo = 9.96; (middle row) Re = 378.79 and Wo = 7.71; and (lower row) Re = 151.51 and Wo = 7.71 at various time instants during the cycle: (a) *t*/*T* = 0.21, (b) *t*/*T* = 0.24, (c) *t*/*T* = 0.27, (d) *t*/*T* = 0.29, (e) *t*/*T* = 0.44, and (f) *t*/*T* = 0.75.

In this section, the effect of Womersley and Reynolds numbers on the flow patterns and vortical structures of simplified sidewall IAs is investigated.  Figure 4 shows the time evolution of the nondimensional out-of-plane vorticity and in-plane velocity vectors for simplified sidewall IAs with different Re and Wo. In Figure 4, the dominant flow patterns are similar for different Re and Wo. In all cases a vortex ring, denoted as R1, starts to form from the proximal wall in the early acceleration phase (Figure 4(a)). Before the inlet velocity reaches the peak systole, the vortex ring is convected across the IA neck (Figures 4(b)–4(d)). Eventually it hits the distal wall and diffuses just after the peak systole and in the deceleration phase (Figures 4(e) and 4(f)). A similar behavior can be observed in Figure 5, which shows the time evolution of the three-dimensional vortical structures visualized by the isosurface of *q*-criteria for simplified sidewall IAs with different Re and Wo. In all cases the vortex ring forms at early acceleration phase (Figure 5(a)) and is convected across the IA neck (Figures 5(b)–5(d)) and hits the distal wall before the peak systole phase. Subsequently, it rolls up to form a recirculation vortical structure, denoted as VS1, which stays until the end of the cycle. The size of vortex ring for all cases is approximately similar (Figure 5). The An for all the simplified sidewall cases is equal to 1.43, which is consistent with the previous work [11, 17, 26] stating that when An > 1 the flow is dominated by the vortex formation.

The only difference in the vortex ring formation and evolution is the location of the vortex ring for different Wo and Re (Figures 4 and 5). To further investigate the effect of Re and Wo, the location of the center of the vortex ring at different time instants in the cycle is identified by the maximum *q*-criteria on the midplane of simplified sidewall IAs and is shown by the green crosses in Figure 4. As it can be observed by comparing different rows of Figures 4(a)–4(d), the location of vortex ring is different at specific time instants. The effect of Wo on the convection of the vortex ring can be observed by comparing middle and upper rows of Figures 4(a)–4(d) at specific time instants; that is, the convection of the vortex ring corresponding to Wo = 7.71 (middle row) is higher than Wo = 9.96 (upper row) with similar Re = 378.79 because the distance of vortex ring from proximal wall is higher for Wo = 7.71 (middle row) at similar *t*/*T* in the cycle. The effect of Re on the convection of the vortex ring can be observed by comparing middle and lower rows of Figures 4(a)–4(d) at specific time instants; that is, the convection of the vortex ring corresponding to Re = 378.79 (middle row) is higher than Re = 151.51 (lower row) with similar Wo = 7.71. This behavior can be observed by the location of the nondimensional out-of-plane vorticity as well. The convection of the vortex ring in simplified sidewall IAs and its effect on hemodynamic stresses is discussed in detail in Sections 4.4 and 4.5, respectively.

### 4.2. Bifurcation IA Simplified Models

**Figure 6:**
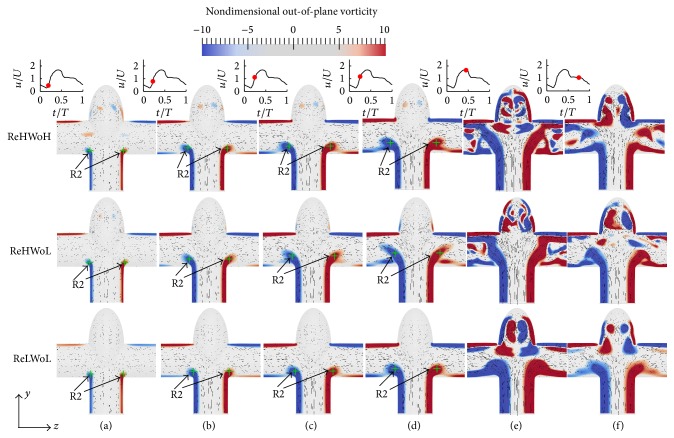
Evolution of the nondimensional out-of-plane vorticity and in-plane velocity vectors for simplified bifurcation IAs with different Re and Wo; (upper row) Re = 378.79 and Wo = 9.96; (middle row) Re = 378.79 and Wo = 7.71; and (lower row) Re = 151.51 and Wo = 7.71 at various time instants during the cycle: (a) *t*/*T* = 0.19, (b) *t*/*T* = 0.22, (c) *t*/*T* = 0.24, (d) *t*/*T* = 0.25, (e) *t*/*T* = 0.44, and (f) *t*/*T* = 0.75. R2 depicts the vortex ring in simplified bifurcation IAs and the green cross shows the location of the maximum *q*-criteria on the midplane of IAs.

In this section, the effect of Womersley and Reynolds numbers on the flow patterns and vortical structures of simplified bifurcation IAs is investigated.  Figure 6 shows the time evolution of the nondimensional out-of-plane vorticity and in-plane velocity vectors for simplified bifurcation IAs with different Re and Wo. From Figure 6, the dominant flow patterns, that is, vortex formation, are similar in all simulations of simplified bifurcation IAs. This is consistent with our previous simulations with An > 1 in terms of vortex formation. The vortex ring, denoted as R2, starts to form at inlet/outlet cross sections in the early acceleration phase (Figure 6(a)). Subsequently, the vortex ring grows in size, while it moves to the outlets (Figures 6(b)–6(d)). Finally in the peak systole, the complicated vortical structures are observed (Figure 6(e)).

**Figure 7:**
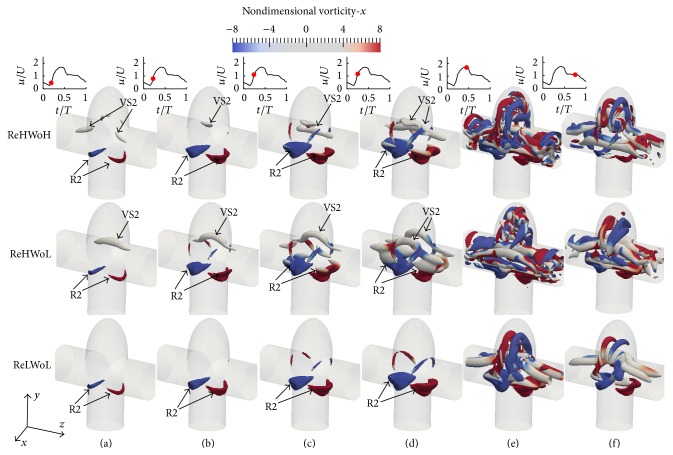
Evolution and topology of the three-dimensional vortical structures colored by vorticity-*x* magnitude for simplified bifurcation IAs with different Re and Wo; (upper row) Re = 378.79 and Wo = 9.96; (middle row) Re = 378.79 and Wo = 7.71; and (lower row) Re = 151.51 and Wo = 7.71 at various time instants during the cycle: (a) *t*/*T* = 0.19, (b) *t*/*T* = 0.22, (c) *t*/*T* = 0.24, (d) *t*/*T* = 0.25, (e) *t*/*T* = 0.44, and (f) *t*/*T* = 0.75.

At peak systole more segments of vortical structures in the dome can be observed for the higher Re (middle and upper rows of Figure 6(e)) in comparison to the lower Re (the lower row of Figure 6(e)). This is the consequence of the vortex ring interactions with other vortical structures, which cannot be seen in two-dimensional vorticity on the midplane. In order to show the three-dimensional vortical structures, Figure 7 plots the time evolution of the three-dimensional vortical structures visualized by the isosurfaces of the *q*-criteria for simplified bifurcation IAs with different Re and Wo. Other than the vortex ring, another vortical structure can be seen at the inlet/outlet cross section close to the IA neck, denoted as VS2, for Re = 387.79 (middle and upper rows in Figures 5(a)–5(d)). VS2 is observed by Vigolo et al. [39] for the first time and later by Chen et al. [40] in *T*-junction pipes and named “particle trapping vortical structures.” Particle trapping vortical structures are distinguished from other structures by the vorticity magnitude in normal direction to the midplane of models (*x*-direction) in Figure 7, because particle trapping structures have almost zero vorticity in the normal direction to the midplane of the models. Because of the existence of an IA in the current study, the particle trapping is distorted in comparison to that in the *T*-junction pipe [39].

One effect of Re in simplified bifurcation IAs is that particle trapping vortical structures do not form at the acceleration phase of Re = 151.51 (the lower row of Figure 7). This behavior is also observed by Vigolo et al. [39] who reported that particle trapping vortical structures do not form for Re < 200. As a consequence, more complex and highly asymmetric flow patterns are observed for Re = 387.79 (upper and middle row of Figure 7(e)) in contrast to Re = 151.51 because of the vortex ring interaction with the particle trapping vortical structure.

The green crosses in Figure 6 show the location of the center of the vortex ring by the maximum *q*-criteria on the midplane of simplified bifurcation IAs at different time instants in the cycle. The effect of Wo on the convection of the vortex ring can be observed by comparing middle and upper rows of Figures 6(b)–6(d) at specific time instants; that is, the convection of the vortex ring corresponding to Wo = 7.71 (middle row) is higher than Wo = 9.96 (upper row) at similar Re = 378.79 and *t*/*T*. The effect of Re on the convection of the vortex ring can be observed by comparing middle and lower rows of Figures 6(b)–6(d) at specific time instants; that is, the convection of vortex ring corresponding to Re = 378.79 (middle row) is higher than Re = 151.51 (lower row) with similar Wo = 7.71 at the same *t*/*T*. This behavior is similar to what was observed in sidewall IAs in the previous section (Figure 4). The convection of the vortex ring in simplified bifurcation IAs and its effect on hemodynamic stresses is discussed in detail in Sections 4.4 and 4.5, respectively.

### 4.3. Patient-Specific IA

**Figure 8:**
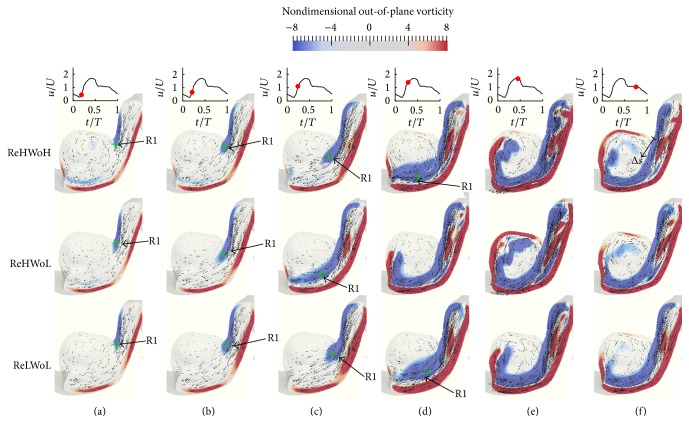
Evolution of the nondimensional out-of-plane vorticity and in-plane velocity vectors for patient-specific IAs with different Re and Wo; (upper row) Re = 363.64 and Wo = 9.56; (middle row) Re = 363.64 and Wo = 7.4; and (lower row) Re = 145.45 and Wo = 7.4 at various time instants during the cycle: (a) *t*/*T* = 0.19, (b) *t*/*T* = 0.22, (c) *t*/*T* = 0.24, (d) *t*/*T* = 0.25, (e) *t*/*T* = 0.44, and (f) *t*/*T* = 0.75. R1 depicts the vortex ring in patient-specific IAs and the green cross shows the location of the maximum *q*-criteria on the midplane of IAs.

**Figure 9:**
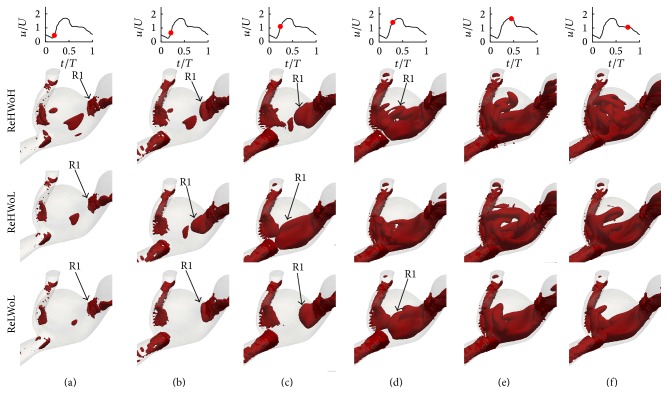
Evolution and topology of the three-dimensional vortical structures for patient-specific IAs with different Re and Wo; (upper row) Re = 363.64 and Wo = 9.56; (middle row) Re = 363.64 and Wo = 7.4; and (lower row) Re = 145.45 and Wo = 7.4 at various time instants during the cycle: (a) *t*/*T* = 0.19, (b) *t*/*T* = 0.22, (c) *t*/*T* = 0.24, (d) *t*/*T* = 0.25, (e) *t*/*T* = 0.44, and (f) *t*/*T* = 0.75.

In this section, the effect of Womersley and Reynolds numbers on the flow patterns and vortical structures of a patient-specific IA, which is of sidewall type, is investigated.  Figure 8 shows the time evolution of the nondimensional out-of-plane vorticity and in-plane velocity vectors for patient-specific IAs with different Re and Wo. The plane is selected the way that vortical structures in the dome can be observed clearly (the plane is shown in Figure 2(b)). From Figure 8, the dominant flow patterns are similar to sidewall IAs (Figure 4). In all cases with different Re and Wo a vortex ring, denoted as R1, starts to form from the proximal wall in the early acceleration phase (Figure 8(a)). The vortex ring evolves as it is convected across the IA neck (Figures 8(b)–8(d)). Finally it hits the distal wall and diffuses after the peak systole and in the deceleration phase (Figures 8(e) and 8(f)). A similar behavior can be observed from Figure 9 for different Re and Wo, which shows the time evolution of the three-dimensional vortical structures visualized by the isosurface of *q*-criteria for patient-specific IAs. In all cases the vortex ring forms at early acceleration phase (Figure 9(a)) and is convected across the IA neck (Figures 9(b)–9(d)) and hits the distal wall before the peak systole phase. An for all cases is equal to 2.4, which is consistent with the previous work [11, 17, 26] stating that when An > 1 the flow is dominated by the vortex formation. The only effect of Wo and Re on the vortex ring formation and evolution is the location of the vortex ring at specific time (Figures 8 and 9).

By comparing the flow structures in patient-specific IAs (Figure 9) with simplified sidewall IAs (Figure 5), it can be observed that more complex and broken vortical structures are formed in the patient-specific IA in comparison to the simplified sidewall IA, while the dominant flow pattern (vortex ring formation and evolution) is similar. The vortex ring in patient-specific IA is deformed because of the complexity of its geometry.

To further investigate the effect of Re and Wo, the location of the center of the vortex ring at different time instants in the cycle is identified by the maximum *q*-criteria on the plane (shown in Figure 2(b)) of patient-specific IAs and is shown by the green crosses in Figure 8. The similar behavior with simplified models (Figures 4 and 6) can be observed in the patient-specific geometry (Figure 8); that is, the convection of the vortex ring corresponding to Wo = 7.4 (middle row) is higher than Wo = 9.56 (upper row) with similar Re = 363.64 because the distance of vortex ring from proximal wall is higher for Wo = 7.4 (middle row) at similar *t*/*T* in the cycle. The convection of the vortex ring corresponding to Re = 363.64 (middle row) is higher than Re = 145.45 (lower row) with similar Wo = 7.4. The convection of the vortex ring in the patient-specific IAs and its effect of hemodynamic stresses are discussed in detail in Sections 4.4 and 4.5, respectively.

### 4.4. The Effect of Re and Wo on the Location of the Vortex Ring

**Figure 10:**
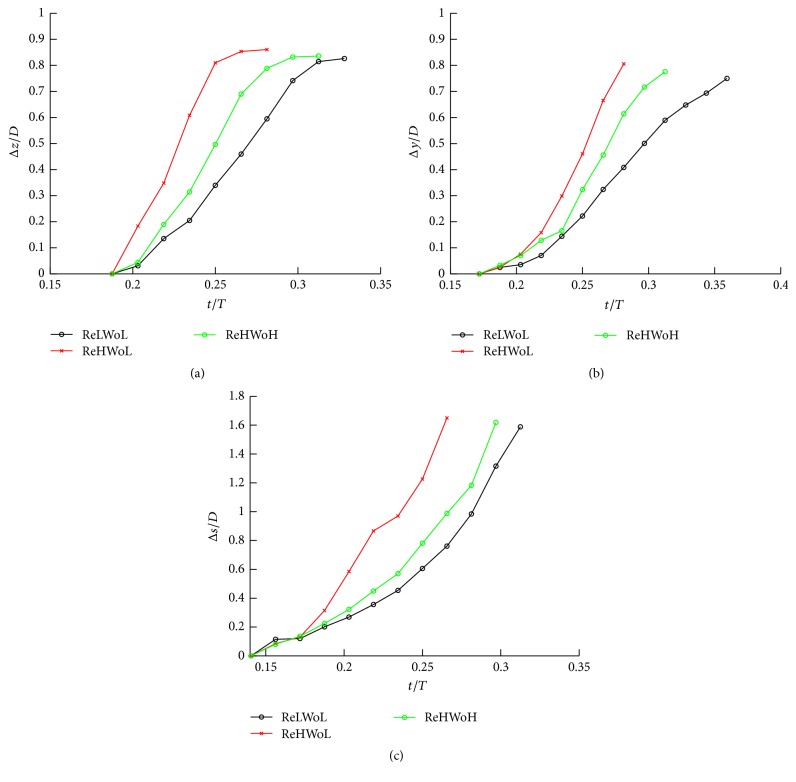
The distance of the maximum *q*-criteria [35] on the midplane for different Re and Wo for (a) sidewall IAs, where the vortex ring distance is measured from the proximal wall in the *z*-direction; (b) bifurcation IAs, where the vortex ring distance is measured from the inlet/outlet cross section in *y*-direction; (c) patient-specific IAs, where the vortex ring distance is measured from the proximal wall in the *s*-direction at various time instants during a part of cycle that vortex ring forms and breaks down. This distance is normalized by the IA width (*D*), distance between proximal and distal wall (*W*), and outlet diameter (*D*) in simplified sidewall, patient-specific, and simplified bifurcation IAs, respectively. Values for ReHWoH, ReHWoL, and ReLWoL can be found in Table 1 for different geometries.

In this section, the effect of Wo and Re on the convection of the vortex ring in simplified IAs (sidewall and bifurcation) and a patient-specific IA is investigated, and a formula for the location of the center of the vortex ring is derived using dimensional analysis.  Figure 10 plots the distance of the maximum *q*-criteria (location of the center of the vortex ring) on the midplane of simplified IAs (sidewall and bifurcation) and the plane of patient-specific IAs at different Re and Wo. These distances (Δ*s*) are calculated from the proximal wall for sidewall (in *z*-direction shown in Figure 1(a)) and also from the proximal wall for patient-specific IAs (in *s*-direction shown in upper row of Figure 8(f)) and from the inlet/outlet cross section for bifurcation IAs (in *y*-direction shown in Figure 1(b)). Δ*s* is normalized by the diameter of the inlet parent artery (*D*) in all test cases. It can be observed that the vortex ring formation starts in *t*/*T* ≈ 0.19 and 0.17 for simplified sidewall and bifurcation IAs, respectively, regardless of Re and Wo. The location of the vortex ring is different in various Re and Wo at similar instants in the cycle. Based on Figure 10 and previous discussion in Figures 4, 6, and 8, high Re and low Wo are associated with faster convection of the vortex ring since the vortex ring corresponding to high Re and low Wo reaches the distal wall sooner. This behavior can be explained by dimensional analysis. The dimensionless distance of the vortex ring from the proximal wall can be defined as

(3) $$\begin{array}{c} \frac{\Delta s}{D}=\frac{ut}{D}, \end{array}$$

where *u* and *t* are the vortex convection velocity and time, respectively. Δ*s* is the distance of the vortex ring location from the proximal wall in simplified sidewall IAs (Δ*z*), also the proximal wall in patient-specific IAs (Δ*s*), and the distance of the vortex ring location from the inlet/outlet cross section in bifurcation IAs (Δ*y*) as denoted in Figure 1. Here, we simply approximate the vortex ring convection velocity by assuming that it is equal to the bulk flow velocity in the parent artery. Applying the dimensional analysis in (3) leads to

(4) $$\begin{array}{c} \frac{\Delta s}{D}=\bar{u}\bar{t}\frac{UT}{D}, \end{array}$$

where $\bar{u}=u/U$ and $\bar{t}=t/T$. Re = *UD*/*ϑ* and Wo^2^ = *D* ^2^2*π*/*Tϑ* definitions can be applied in (4), which results in

(5) $$\begin{array}{c} \frac{\Delta s}{D}=2\pi \bar{u}\bar{t}\frac{Re}{Wo^{2}}. \end{array}$$

Based on (5), decreasing Re and increasing Wo result in decreasing distance of the vortex ring from the proximal wall at specific instants ($\bar{t}$), which is in agreement with Figure 10. It is noted that $\bar{u}$( = *u*/*U*) is the dimensionless velocity, which is similar for all simulations at the specific dimensionless time (*t*/*T*) regardless of Re and Wo (as can be seen from Figure 3). The effectiveness of Re and Wo on the normalized distance of the vortex ring from the proximal wall can be compared using (5) as follows:

(6) ΔsRe=378.79,Wo=7.71ΔsRe=378.79,Wo=9.96=1.67,ΔsRe=378.79,Wo=9.96ΔsRe=151.51,Wo=7.71=1.5.

The trend observed in (6) agrees well with that of Figure 10, meaning that Δ*s*_(ReHWoL)_ > Δ*s*_(ReHWoH)_ and Δ*s*_(ReHWoH)_ > Δ*s*_(ReLWoL)_ at specific instants (*t*/*T*). The trend of the location of the vortex ring for various Re and Wo agrees well with the formulation developed from experiments on thin core rings generated by a piston gun in water [41]. By substituting simulation specifications of current study on the formulations expressed in [41] Δ*s*_(Re = 378.79, Wo = 7.71)_/Δ*s*_(Re = 378.79, Wo = 9.96)_ ≈ 1.62 and Δ*s*_(Re = 378.79, Wo = 9.96)_/Δ*s*_(Re = 151.51, Wo = 7.71)_ ≈ 1.73 can be reached which shows promising agreement with the simple formulation obtained in this study (6), considering that in [41] the vortex forms in the tank instead of an IA neck.

### 4.5. The Effect of Re and Wo on the Oscillatory Shear Index and Wall Shear Stress

**Figure 11:**
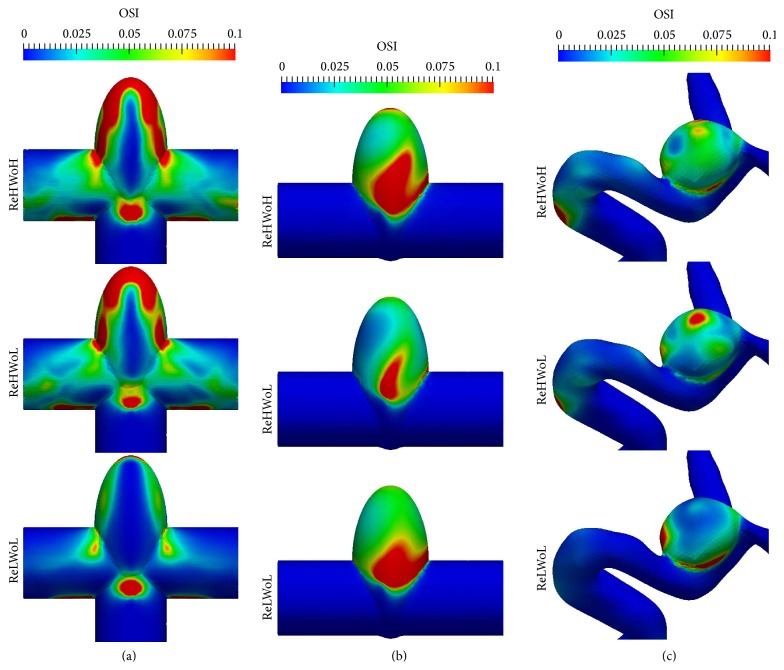
Cycle-averaged distribution of oscillatory shear index with different Re and Wo for (a) simplified bifurcation, (b) simplified sidewall, and (c) patient-specific IAs. The oscillatory shear index is calculated based on the cycle-averaged OSI. Values for ReHWoH, ReHWoL, and ReLWoL can be found in Table 1 for different geometries.

**Figure 12:**
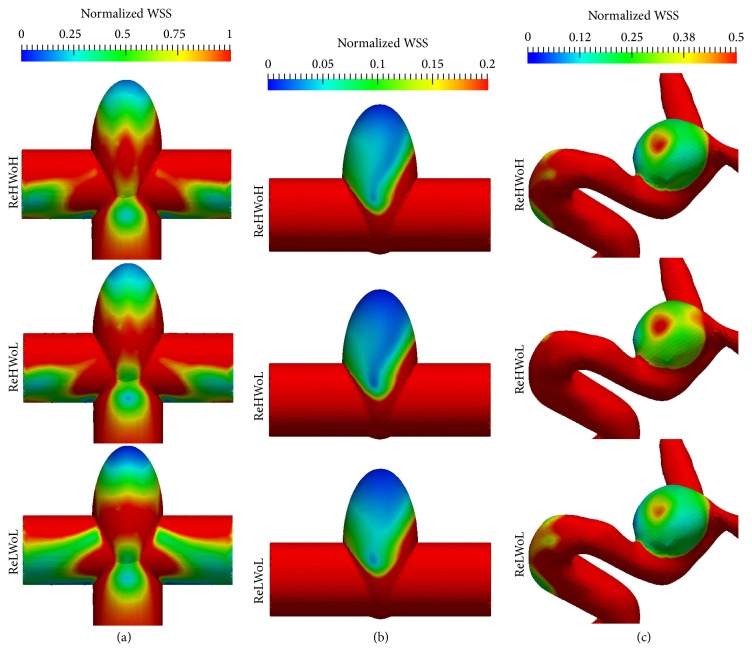
Cycle-averaged distribution of normalized wall shear stress with different Re and Wo for (a) simplified bifurcation, (b) simplified sidewall, and (c) patient-specific IAs. WSS is normalized by that of the inlet parent artery. Values for ReHWoH, ReHWoL, and ReLWoL can be found in Table 1 for different geometries.

**Figure 13:**
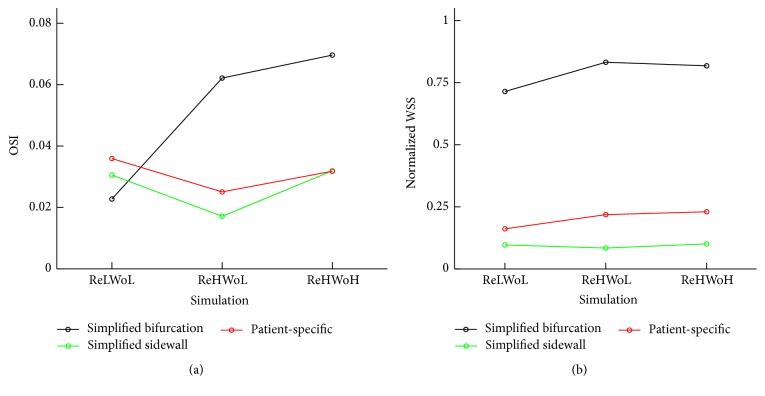
(a) Oscillatory shear index and (b) normalized wall shear stress for different Re and Wo at simplified bifurcation, simplified sidewall, and patient-specific IAs. WSS and OSI are averaged on the dome surface. The resulting WSS is normalized by that of the parent artery. Values for ReHWoH, ReHWoL, and ReLWoL can be found in Table 1 for different geometries.

The oscillatory shear index (OSI) and wall shear stress (WSS) are important parameters in determining aneurysm rupture risk [2, 42, 43]. In this section, we investigate the effect of Re and Wo on OSI and WSS in terms of the dynamic behavior of the vortex ring. Figures 11 and 12 show the cycle-averaged distribution of oscillatory shear index and normalized wall shear stress, respectively, with different Re and Wo (Table 1) for (a) simplified bifurcation, (b) simplified sidewall, and (c) patient-specific IAs. For the purpose of comparison, WSS of the IA dome is normalized by that of the inlet parent artery similar to previous work [2]. To quantify the effect of Re and Wo on the hemodynamic stresses, normalized WSS and OSI are averaged over the dome surface. The results are plotted in Figure 13, which shows oscillatory shear index and normalized wall shear stress for different Re and Wo on simplified bifurcation, simplified sidewall, and patient-specific IAs.

It can be observed in Figure 13(a) that in the simplified sidewall IA OSI for low Re (lower row of Figure 11) is 79% higher than that of high Re (middle row of Figure 11) with similar Wo. In addition, OSI for high Wo (upper row of Figure 11) is 86% higher than that of low Wo (middle row of Figure 11) with similar Re in the simplified sidewall IA. A similar trend can be seen in the patient-specific IA for OSI; that is, OSI for low Re (lower row of Figure 11) is 43% higher than that of high Re (middle row of Figure 11) with similar Wo and OSI for high Wo (upper row of Figure 11) is 27% higher than that of low Wo (middle row of Figure 11) with similar Re. This is due to the fact that low Re and high Wo increase the vortex residence time (the time that vortex ring hits the wall and breaks down minus the time that vortex ring starts to form, normalized by the period of the cycle) in the dome of sidewall IAs (Figures 10(a) and 10(c)), which increases the flow disturbance in the cycle and, consequently, results in higher OSI.

For the simplified bifurcation IA, in contrast to sidewall IAs, it can be observed in Figure 13(a) that OSI corresponding to Wo = 9.96 is slightly (12%) higher than Wo = 7.71 with similar Re = 378.79. OSI corresponding to Re = 151.51 is 63% lower than Re = 378.79 in simplified bifurcation IA with similar Wo = 7.71 because of the absence of “particle trapping” vortical structures at acceleration phase of Re = 151.51, which decreases the segments of vortical structures (by comparing lower and middle row of Figure 7) and flow disturbance.

It can be observed in Figure 13(b) that Wo and Re variations change the normalized WSS by only a maximum of 14% among all IAs. Therefore, we conclude that the effect of Wo and Re variations on the normalized WSS is severalfold smaller than that of OSI.

## 5. Conclusions

The effect of two key parameters, that is, Reynolds and Womersley number, on the hemodynamics of simplified IAs (sidewall and bifurcation) and patient-specific IAs is investigated using CFD simulations. Based on our results, the dominant flow pattern and the vortex structure, for example, the vortex ring formation, remain similar by modifying Re from 378.79 to 151.51 and Wo from 7.71 to 9.96 in both simplified bifurcation and sidewall IAs. Similarly, the vortex ring formation remains similar by modifying Re from 363.64 to 145.45 and Wo from 7.4 to 9.56 in patient-specific IAs. However, the location of the vortex ring at different instants in a cycle in both simplified and patient-specific IAs is controlled by Wo and Re. In addition, the interaction of the vortex ring with other vortical structures depends on Re in bifurcation IAs.

The location of vortex ring as function of time depends on the combination of Re and Wo. We found that the high Re and low Wo are associated with the fast convection of the vortex ring in IAs. Using dimensional analysis, a formulation (Equation (5)) is obtained which can clearly demonstrate the trend of vortex ring distance from the proximal wall in simplified sidewall and patient-specific IAs and from the inlet/outlet cross section of simplified bifurcation IAs. Based on the obtained formula, the location of vortex ring is proportional to Re/Wo^2^ which shows good agreement with formulations expressed in [41] for the vortex ring location in a tank.

The highly asymmetric and complicated vortical structure observed in bifurcation IAs at Re = 378.79 in comparison to Re = 151.51 are a consequence of the interaction between the vortex ring and the particle trapping vortical structures. More organized vortical structures are observed for Re = 151.51 since the particle trapping vortical structure does not form in the acceleration phase at low Re (Re < 200 according to [39]). Therefore, there is no interaction between the vortex ring and the particle trapping vortical structures in bifurcation IAs with Re = 151.51.

We found that variations of Wo and Re slightly affect the normalized WSS (maximum of 14%), while OSI is proportional to Wo and 1/Re in sidewall IAs (both simplified and patient-specific). The observed trend of OSI in these IAs is a consequence of higher residence time of the vortex (because the vortex reaches the distal wall later in the cycle at high Wo and low Re), which disturbs the flow for a longer time in each cycle and increases the OSI. This trend is not observed in bifurcation IAs because the ring vortex is convected toward the outlets and does not enter the dome. The shear stress in bifurcation IAs is dominated by the particle trapping structures. In fact, the observed lower OSI (63%) in bifurcation IAs at Re = 151.51 in comparison to Re = 378.79 is because of the absence of particle trapping structures at Re = 151.51. Increasing Wo from 7.71 to 9.96 only slightly increased OSI (12%) at similar Re = 378.79 in simplified bifurcation IAs.

Based on our results, the hemodynamics of the simplified and the patient-specific sidewall IAs is similar in terms of vortex formation, propagation, normalized WSS, and OSI. The bifurcation IA showed different OSI trend relative to the sidewall ones. Note that the conclusions of this work are based on two idealized geometries and on two idealized and one patient-specific geometries for two Re and Wo. We believe our results are valid for other geometries in the physiological range of Re and Wo for aneurysms. Nevertheless, this will be tested in a cohort of aneurysms rigorously in the future.

## Figures and Tables

**Figure 14:**
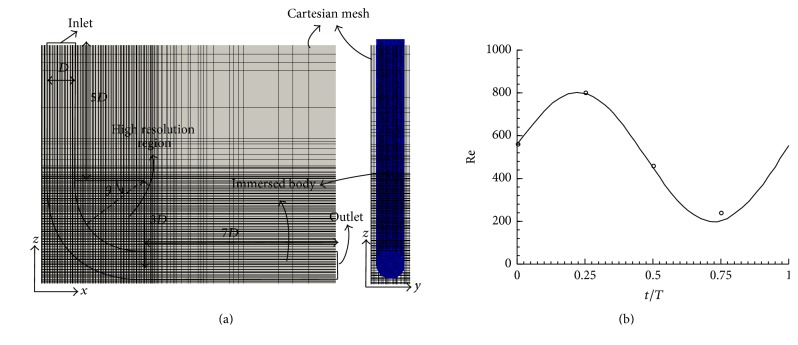
(a) The geometry for the 90° bend (b) incoming flow waveform. Solid line: analytical nondimensional bulk velocity waveform prescribed at the inlet of the flow domain obtained by solving the Womersley equation; circles: the measurements of nondimensional inlet bulk velocity from experiments [31]. The immersed body is meshed with triangular elements.

**Figure 15:**
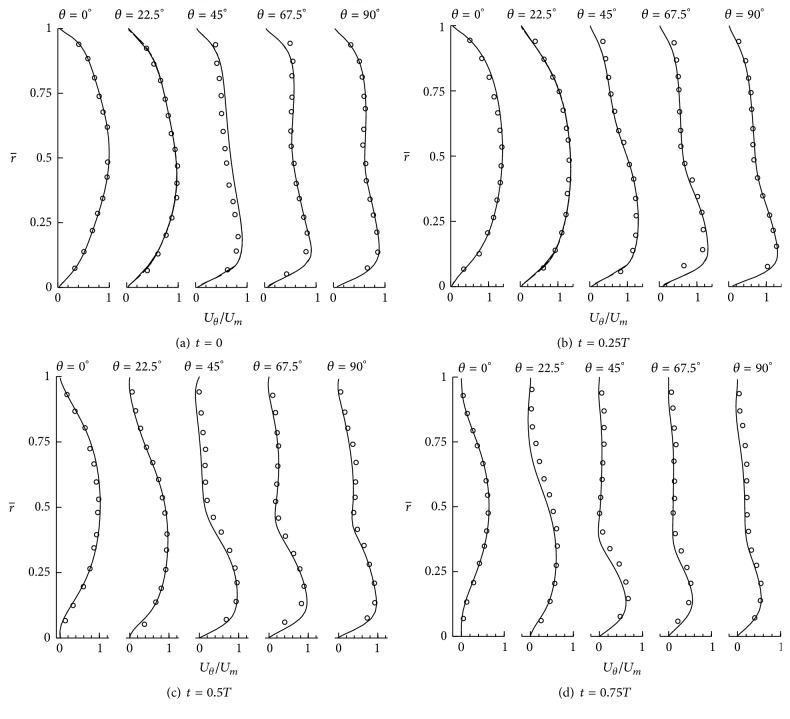
Pulsatile flow through a 90° bend. The calculated streamwise velocity profiles at the plane of symmetry are compared with experimental results (circles) [31] at different time instants. $\bar{r}=(r-r_{\circ })/(r_{i}-r_{\circ })$ (*r*_∘_ and *r*_*i*_ are the outer and inner bend radius, resp.), *U*_*θ*_ is the streamwise velocity at the symmetry plane, and *U*_*m*_ is the maximum streamwise velocity at the symmetry plane at *t*/*T* = 0 and *θ*° = 0.

**Table 1:** Specifications of simulations. H, high; L, low; *D*, diameter; *T*, heart beat period; Re, Reynolds number; Wo, Womersley number; *U*, the average bulk inlet velocity; An, aneurysm number; $\Delta \overset{-}{t}$, the nondimensional time-step.

Simulation	IA type	*D* (m)	Heart beats per minute	*T* (s)	Wo	*U* (m/s)	Re	An	$\Delta \overset{-}{t}$
ReLWoL	Simplified sidewall	0.005	75	0.8	7.71	0.1	151.51	1.43	0.002
ReHWoL	Simplified sidewall	0.005	75	0.8	7.71	0.25	378.79	1.43	0.005
ReHWoH	Simplified sidewall	0.005	125	0.48	9.96	0.25	378.79	1.43	0.003
ReLWoL	Simplified bifurcation	0.005	75	0.8	7.71	0.1	151.51	2.86	0.002
ReHWoL	Simplified bifurcation	0.005	75	0.8	7.71	0.25	378.79	2.86	0.005
ReHWoH	Simplified bifurcation	0.005	125	0.48	9.96	0.25	378.79	2.86	0.003
ReLWoL	Patient-specific	0.0048	75	0.8	7.4	0.1	145.45	2.4	0.002
ReHWoL	Patient-specific	0.0048	75	0.8	7.4	0.25	363.64	2.4	0.005
ReHWoH	Patient-specific	0.0048	125	0.48	9.56	0.25	363.64	2.4	0.003

## Appendix

### Validation: Pulsatile Flow through a 90° Bend

The three-dimensional pulsatile flow through a strongly curved 90° pipe bend is simulated to validate our method, which is used to simulate the test cases in this study, for a pulsatile flow inside an immersed body. The geometry of the test case is shown in Figure 14(a). As shown in Figure 14(a), the radius of the curvature of the bend is three times the pipe diameter (*D*). The outlet is placed 7*D* after the bend and the inlet is placed 5*D* before the bend. A gear pump providing a steady flow with the Reynolds number equal to 500 (Re = *UD*/*ϑ*, where *U* is the bulk velocity, *D* is the pipe diameter, and *ϑ* is the kinematic viscosity) in conjunction with a piston pump generating sinusoidal flow waveform with Re = −300 to Re = 300 was used to generate the pulsatile flow waveform in the experiments [31]. The resulting Womersley number of the flow in the experiments was 7.8.

To generate a waveform that matches the experiment's waveform, the inlet boundary condition is the velocity profile set from the Womersley solution of a fully developed pulsatile flow in a circular pipe as follows [44]:

(A.1) uinlet=21−rR2−ikωeiωt1−J0r−iω/vJ0R−iω/v,

where *J*_0_ is the zero-order Bessel function of the first kind, *R* is the radius of the pipe, *r* is the radial distance from the center of the pipe, *ω* = 13.31 rad/s is the angular frequency of the flow oscillation, and *v* is the flow viscosity. The constant *k* is selected to be 0.375 to generate a sinusoidal flow waveform, which matches the experiment's waveform [31, 44]. The above equation is solved using MATLAB, and the resulting solutions are stored and fed into the solver to specify the time-varying inlet flow (Figure 14(b)). As shown in Figure 14(b), the computed inlet Re waveform is in reasonably good overall agreement with the experimental inlet Re. The outlet boundary condition is the Neumann boundary condition. The time period of inflow oscillations, which is nondimensionalized based on *D* and *U*, is *T* = 12.3. The nondimensional time-step Δ*t* = 0.0123 is used for the simulations. The Cartesian domain is discretized by 249 × 53 × 233 grid nodes. The region around the 90° bend is discretized by 179 × 53 × 179 uniform grid nodes and the grid is stretched to the boundaries.

The simulated flow in the 90° bend with the pulsatile inlet is compared with the experimental results [31] in Figure 15. The streamwise velocity profiles at the symmetry plane are plotted at five different locations (i.e., *θ* = 0°, 22.5°, 45°, 67.5°, and 90°) for four different time instants during the cycle (*t* = 0, 0.25*T*, 0.5*T*, and 0.75*T*). The results in Figure 15 are in excellent agreement with the computational results of the same setup with body-fitted grids [20]. Furthermore, as observed in Figure 15, the computational results are in good overall agreement with the experimental results. The maximum discrepancy is at *t* = 0.75*T*, where the largest deviation from experimental inlet waveform from Womersley inlet waveform exists (Figure 14(b)).
